# A Model for Cell Population Size Control Using Asymmetric Division

**DOI:** 10.1371/journal.pone.0074324

**Published:** 2013-09-05

**Authors:** Mani Hamidi, Eldon Emberly

**Affiliations:** Physics Department, Simon Fraser University, Burnaby, British Columbia, Canada; University of Antwerp, Belgium

## Abstract

In multicellular organisms one can find examples where a growing tissue divides up until some final fixed cell number. Asymmetric division plays a prevalent feature in tissue differentiation in these organisms, where the daughters of each asymmetric division inherit unequal amounts of a fate determining molecule and as a result follow different developmental fates. In some tissues the accumulation or decrease of cell cycle regulators acts as an intrinsic timing mechanism governing proliferation. Here we present a minimal model based on asymmetric division and dilution of a cell-cycle regulator that can generate any final population size that might be needed. We show that within the model there are a variety of growth mechanisms from linear to non-linear that can lead to the same final cell count. Interestingly, when we include noise at division we find that there are special final cell population sizes that can be generated with high confidence that are flanked by population sizes that are less robust to division noise. When we include further perturbations in the division process we find that these special populations can remain relatively stable and in some cases even improve in their fidelity.

## Introduction

There are multiple examples of cell populations with controlled final numbers. The size and the accuracy with which this final population number is reached vary. For instance, the number of cells in the *Caenorhabditis elegans* nervous system reaches precisely 302 in every worm [1] while macroscopic organs of thousands of cells in larger organisms also regulate their size [1], [2]. Even similar cell types can show vastly different lineages, as neuroblasts in Drosophila can generate anywhere from 10 s to 100 s of future neuronal cells (reviewed in [3]). In the proliferation and differentiation of tissue, both extrinsic and intrinsic cues have been found to play critical roles in robust size control of the cell population [2], [4], [5]. Extrinsic cues from the micro environment in which a cell finds itself have been shown to drive differentiation in a variety of stem cells, terminating division, sometimes through triggering apoptosis [3]. However, purely intrinsic or autonomous cues also play a role as a variety of cultured or transplanted stem cells can produce lineages nearly identical to those in the endogenous locations. These intrinsic timers have been shown to arise from temporal cascades of transcription factors (as in many neuroblasts [3], [6]) to the accumulation of cell cycle regulators in oligodendrocytes [7], [8]. Thus in some lineages, as cells divide an internal molecular clock akin to an hourglass dictates when they should exit the cell cycle and enter a quiescent stage [8].

Besides the timing of factors that regulate proliferation, asymmetric division (AD), has been found to play a central mechanism in determining the progression of cell lineages. Asymmetric division is an essential mechanism of division in the differentiation of *Drosophila* and *C. elegans* nervous systems [1], [3], [5], [9]. For example the protein Prospero and several other proteins and mRNAs have been identified to asymmetrically divide and orchestrate neuroblast differentiation in the fly embryo [3], [9]. Asymmetrically partitioning factors can arise by a variety of mechanisms [10]. The first and simplest is through unequal volumes of the resulting daughter cells, where the amounts of molecule will be inherited in proportion to the respective volumes. Active localization to basal or apical portions of the dividing cell is another common strategy. In many of the most well studied systems AD tends to generate an all or none inheritance of the cell fate factor, though situations where reaction-diffusion mechanisms that produce more graded distributions are known [10], [11]. Thus there are a variety of molecular mechanisms by which cell fate factors can be distributed between daughter cells.

To our knowledge however no quantitative models have been suggested for how an intrinsic timing mechanism plus asymmetric division can be parameterized to achieve control over population size. Forward engineering approaches have been used to suggest potential solutions to questions in developmental biology, such as self-organized pattern formation [12], [13]. Here we use such an approach to suggest a potential mechanism for achieving a final population of arbitrary size with single integer accuracy. We propose a feedback-free mechanism involving asymmetric division and dilution of a molecule that governs cell division, to allow for accurate and autonomous set-point control over population size. Such a mechanism may play a part in controlling the cell populations of certain tissues or single cell organisms.

**Figure 1:**
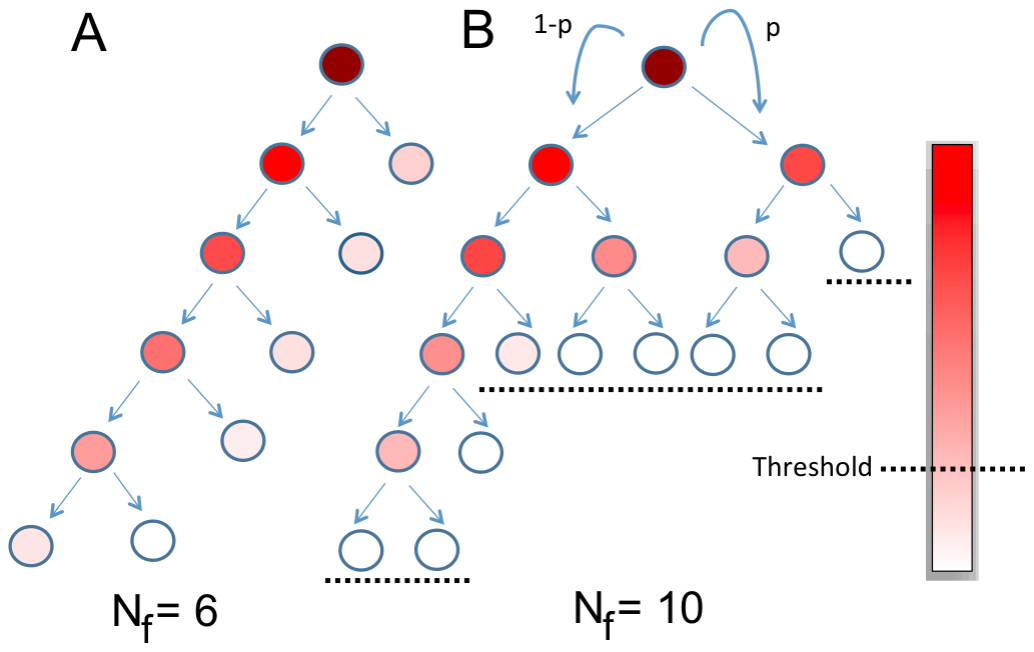
Asymmetric dilution of a growth factor can produce populations of any size. The trees represent growing populations of cells where the color represents the concentration of a growth factor. The parameter 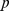 indicates the degree of asymmetry with which the factor is divided between two daughter cells. The threshold (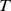) is the concentration of this factor below which the cell can no longer divide. Different final population sizes (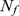) and tree topologies can be achieved by varying 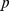 and 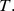 The two trees in this figure demonstrate two such possibilities. (A) A linear topology and (B) non-linear topology.

The quantitative model that we study is based on the suggested hourglass model for the intrinsic timer [8]. In such a mechanism a cell cycle factor is accumulated or diluted with division that ultimately halts the cell cycle when a certain threshold is crossed. We present how such a scheme coupled with asymmetric division can address the problem of reaching an arbitrary controlled final cell count in a growing population. We imagine that the hourglass would be started in the initial progenitor cell via a transient burst of some factor that would then be diluted at each subsequent cell division. In the simplest case of symmetric dilution, the factor will be diluted to 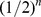
after *n* rounds of division, and therefore given a threshold, 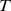 will yield a final population size of 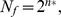 where 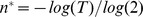 and 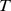 is measured as a fraction of the initial number of molecules present. If asymmetric division is allowed however (Fig. 1), the final population size, 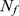, will be dependent on the degree of asymmetry in the growth factor as well as the threshold below which cells can no longer divide, yielding different sizes and topologies of trees (Compare Fig. 1A, B). We show that this model can indeed generate any arbitrary final population size and that there are special population sizes that can be generated with high confidence even in the presence of noise. We discuss how this model may be relevant to the development of certain lineages given the available biological evidence.

## Results

### Deterministic Cell Division and Partitioning

**Figure 2:**
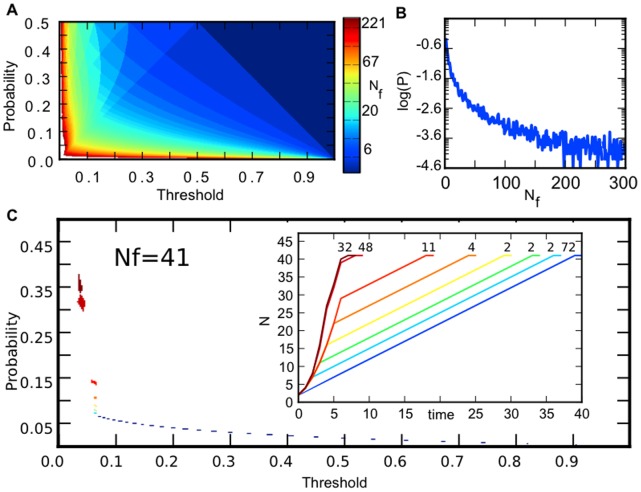
Deterministic simulation of tree growth at all 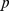 and 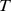
values. (A) 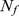 as a function of the degree of asymmetry (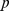) and the threshold (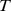). The final population size 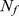 grows as the values of 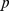 and 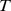 approach zero. (B) The likelihood of finding a parameter combination with a final population size of 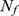 drops rapidly for larger values of 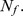 (C) Different and separate regions in parameter space can produce a given 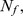 in this case 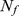 = 41 is shown. The inset and the color-coding indicate that each of these locations produce a tree with a different topology (uniquely identified by its growth curve) despite all of them producing 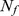 = 41. The numbers above each growth curve indicate the number of parameter pairs that produce that topology.

Using our model we explored whether asymmetric division coupled with dilution of a regulatory molecule could generate an arbitrary final cell count. At each division a fraction of the regulatory molecule, 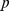, gets put into one cell with the remainder going to the other. When the fraction of protein in a cell gets below a cutoff 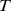, cell division stops. Fig. 2a shows a map of the final cell population size 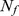 as a function of these two parameters, 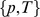. The map shows that all population sizes can be generated using such a scheme from 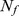 = 2 to any arbitrarily large population size (in the figure we stopped at a maximum 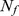 = 310). Small final population sizes have larger areas in parameter space, meaning that there are more combinations of 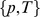 that will generate that size. For instance, the largest area corresponds to 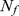 = 2 and this trivially corresponds to 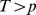 and 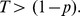 An arbitrary large population size is possible but the area in the 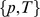 parameter space that generates it gets prohibitively small so as to be unattainable given biochemical mechanisms. These results can be seen in Fig. 2B where we plot the number of 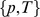 pairs that generated a given final population size. High 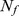 are generated at low threshold cutoff since it requires many more divisions to dilute to this low level. As the division moves from being more asymmetric to symmetric, this threshold needs to get lower in order to generate these high 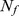 values (as seen on the left border of Fig. 2A). It is also possible to generate high 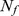 at very low values of 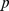 (i.e. highly asymmetric division). Again, now, one cell is getting most of the molecules and will take many rounds of division to dilute.

Given that a final population size has a certain number of parameter combinations, do all parameters yield a similar growth curve? In the inset to Fig. 2C, we show the growth of the population for different 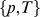 pairs that all yielded a final 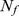 = 41. Some parameters lead to rapid, non-linear growth whereas others generate slow, linear growth curves. Interestingly we find that the two extremes in topology (non-linear and linear) tend to be the most frequent amongst the parameter space for a given final population size (the numbers above each curve represent how many parameters yielded that growth curve). Non-linear growth parameter combinations correspond to higher 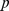 (less asymmetric division) and a low threshold (Fig. 2C upper left), whereas more linear topologies are found at lower 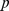 (more asymmetric division) and higher thresholds (Fig. 2c lower right). For these linear growth curves, it is analytically trivial to solve for *some* of the multiple values of 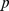
and 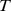 that give any desired 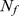. Specifically, for a linear growth curve, 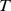 and 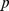 must satisfy: (1) 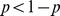 (2) 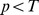 (3) 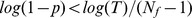 and (4) 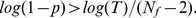 For any arbitrary value of 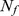 it is guaranteed that a 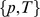 value will exist to produce such a tree. As shown in the inset to Fig. 2C, a variety of topologies exist between non-linear and linear. We find that the variety of different tree topologies grows with the size of the tree, while fewer 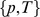
combinations exist for larger 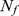 **.** These counteracting forces result in maximal variety of topologies (variety = 11) occurring at ∼ 50 (see Fig. S1). Thus within this asymmetric division model different growth responses are possible that still lead to any arbitrary 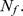 We now consider how such a model responds to the addition of division noise.

### Stochastic Partitioning and Robust Population Sizes

There is inherent stochasticity of molecular segregation at division [14], [15], which was ignored in the simulations above. This variability can confound the fidelity with which a desired 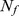 can be achieved; therefore, its effects must be evaluated to assess the viability of this model in natural or synthetic systems. We model this segregation noise as a binomial process, where regulatory molecules in a cell are partitioned at each division with a probability, 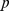, to go to one cell or the other. This assumes independent segregation of the molecules such as might occur where the asymmetry arises purely due to volume differences between the mother and daughter cells. This is a simplifying assumption as other forms of segregation are possible from disordered to ordered segregation [15] where correlations would lead to a departure from purely binomial noise. In these stochastic simulations we now have to introduce a third parameter, namely 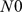, the initial number of molecules in the progenitor cell.

**Figure 3:**
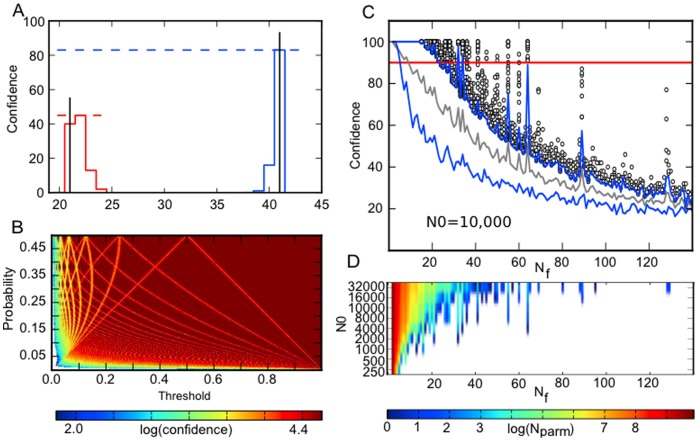
Stochastic simulation of tree growth at all 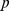 and 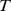
values. (A) Distributions of 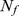 from two different combinations of 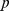 and 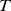 in a stochastic simulation with binomial noise at division. The black lines indicate the resulting 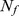 when the same parameter values are used in a deterministic simulation. The shift in the most probable 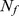 and the probability or confidence with which they occur can vary from parameter to parameter. (B) The confidence of the most probable 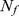
drops in border regions between different 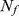 values (compare with Figure 1A) and also at small values of 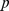 and 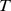 corresponding to high 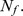 (C) The confidence with which the most probable 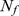 occurs is plotted for all parameter values. Grey dashed line shows the mean, blue lines show one standard deviation from mean, and individual confidences are shown for parameter combinations that resulted in confidences greater than one standard deviation from the mean. Note that despite the rapid drop in the average confidence there exist high (>90%, red line) confidence outliers for 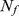 as large as 64. The occurrence of these high confidence outliers appears to be sporadic for 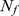 between 32 and 64. Here 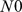 = 10,000. (D) The number of high confidence parameters for any given 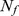 also depends on the value of 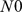, as it prescribes the magnitude of noise at each division. Note that special values of 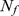 (e.g. 41) contain high confidence parameters for values of 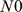 lower than their neighbouring 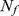 values.

We simulated our stochastic model (see Methods) and calculated the distribution of final cell population sizes for each 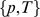
pair with initial starting molecule number of 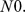 Some of the generated distributions are shown in Fig. 3A. Interestingly there are some 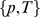 values for which the most probable 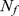 corresponds to that of the deterministic case (see Fig. S3) whereas there are other 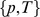 values for which the most probable 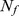 is different than what would have been generated in the deterministic simulation. When 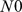 is low, growth is inherently noisier and the difference between the population sizes generated in the deterministic case and the stochastic simulation is stark (see Fig. S2). Not surprisingly, as 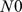 is increased, the stochastic simulation converges toward the deterministic results (see Fig. S2).

We are particularly interested in 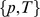 values that generate a population size with high likelihood. In particular, what 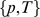 values have their most probable 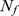 occur >90% of the time? Such 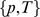 values are insensitive to the noise and yield populations that yield the same number of cells with high confidence (see Fig. 3A). Can all population sizes be generated with high confidence? Or are there some that are more difficult to generate when noise is added? In Fig. 3B we show the probability of the most probable 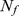 for each 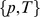 pair. This shows that there are a number of 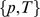 pairs that can generate small 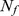 with high confidence. This is not true for larger 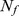 where the number of 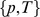 pairs is much smaller, with some having hardly any 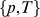 that can yield >90% confidence. Also not surprisingly for those parameters that reside near the transitions between 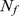 the probability of the most likely 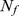 also drops. In Fig. 3C we plot these confidence values against the most probable 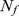 value for all 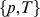 pairs sampled. With this particular 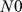, all small population sizes up to 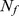 ∼ 20 can be generated with high confidence 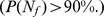 What is remarkable is that at higher 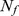 (e.g. 41), there exist *special* population sizes that can also be generated with high confidence, yet sizes that are either smaller or bigger by one are low confidence. This is reminiscent of the emergence of magic numbers in other systems.

We summarize our findings for these special population sizes in Fig. 3D. In this figure we plot the number of parameters that yield a given 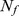 with probability >90% as a function of the starting molecule number. At low 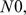 only the smallest of population sizes can be generated with high confidence. Yet as 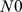 increases, it can be seen that there are larger population sizes that occur with high confidence and these particular 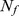
follow a complex pattern of emergence. In the next section we will explore if these special population sizes are robust to perturbations in the division process, more so than other population sizes.

**Figure 4:**
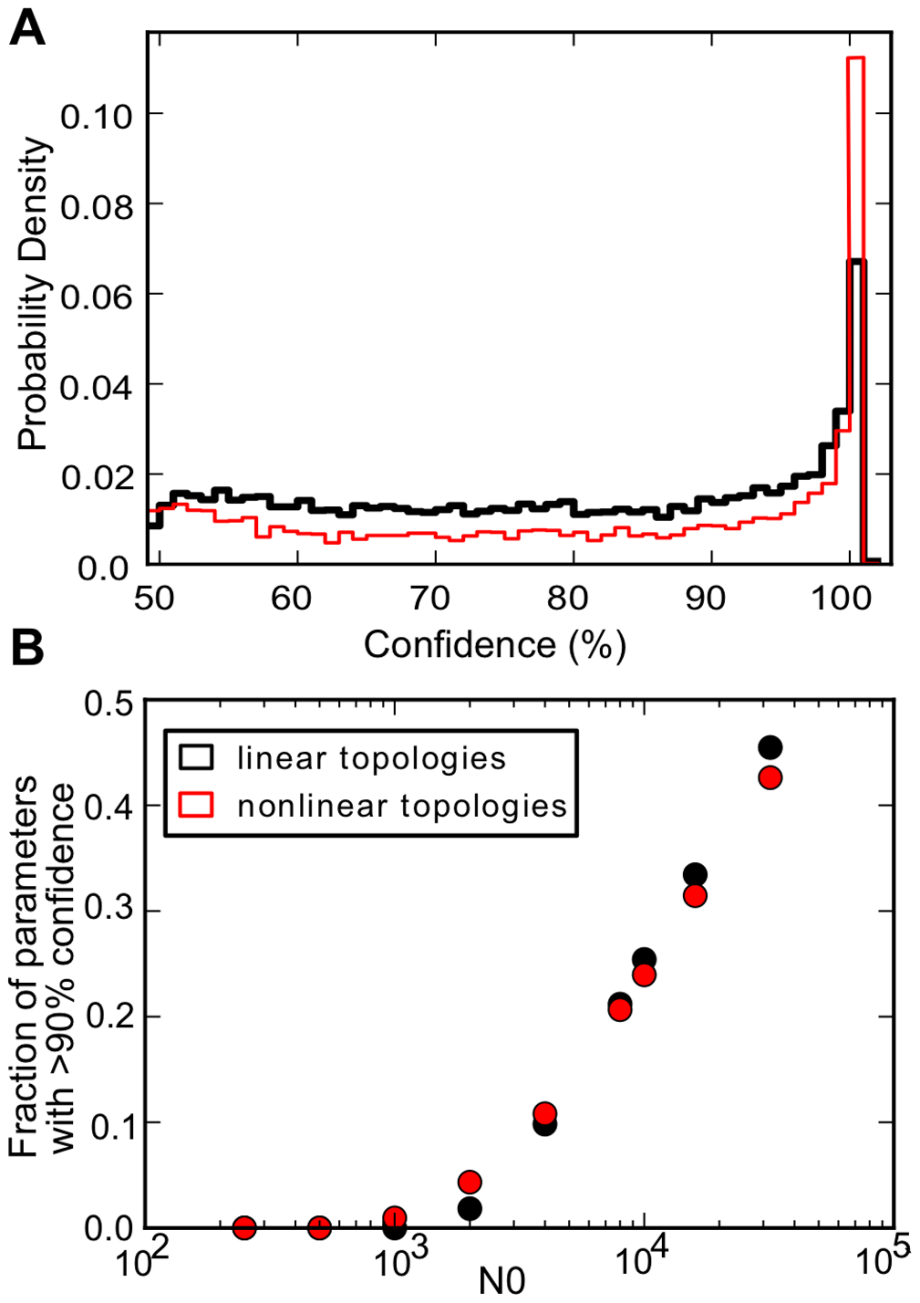
The relationship between tree topology and confidence. (A) For 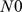 = 10,000, a higher percentage of linear topologies (i.e. linear growth-curve) exhibit a high (>90%) confidence. There are however always more non-linear topologies with 100% confidence. (B) This trend appears to revert at about 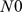 = 8,000. The percentage of high-confidence non-linear topologies is higher for 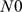 <8,000.

As in the deterministic case, when simulating a given 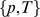 pair stochastically, the resulting growth curves all follow a particular topology. Are there topologies that are more likely to generate a final population size with high confidence? For every 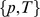 pair we characterized the topology of the growth curve in the stochastic simulation (see Methods). In Fig. 4A we plot the chance that either the linear or non-linear topologies will generate a final population size with the given likelihood. Overall for this value of 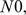 linear topologies tend to generate more high confidence population sizes. In Fig. 4B we show the fraction of high confidence 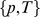 pairs (i.e. the most probable 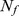 was >90%) for both the linear and non-linear cases as a function of 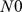 (these results are shown in detail in Fig. S4). Interestingly at low 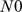, non-linear growth is much better at generating high-confidence 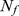 yet at higher 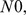 it shifts over to linear growth being the best. Thus topology has a role in determining the confidence with which a final 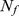 will be generated and non-linear growth is less sensitive when division is noisy and linear growth is better when noise is minimal.

### Sensitivity Analysis of Population Size

In the previous section we added noise at division by assuming that the number of molecules is distributed by some independent random process at each division. However there was no noise in either the value of 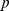 or 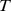. In this section we consider allowing these parameters to be perturbed during the division process. For every dividing cell we allow each parameter, 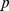 or 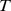 to vary by some amount.

**Figure 5:**
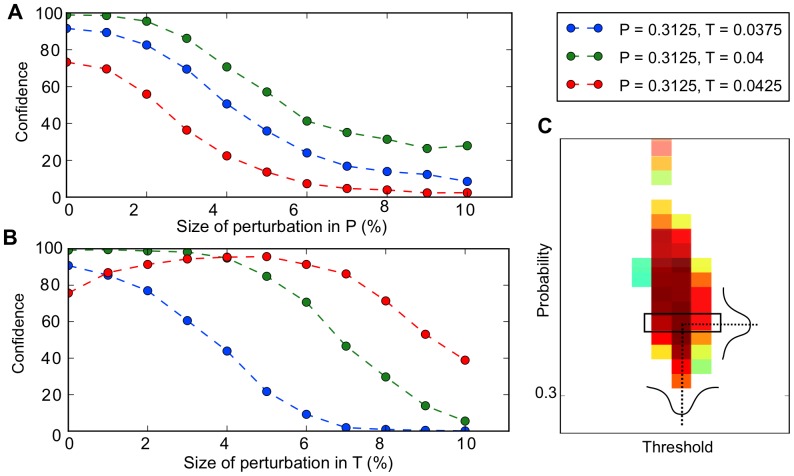
Special values of 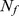 exhibit robustness against perturbations in 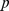 and 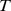. (A) Perturbations in 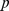 show that parameters with higher initial confidence also exhibit robustness to larger perturbations in 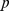. (B) Perturbations in 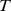 can produce critical behavior where a parameter combination with an initially low confidence can benefit from added noise to the value of 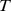. (C) This shows the region in parameter space from which the three parameter combinations were chosen (black box). The colors indicate the initial confidence at that point before perturbation.

In particular we are interested in exploring whether those special population sizes that can be generated with high confidences are less sensitive to parameter perturbations than those which are lower confidence. In Fig. 5 we show the results of perturbing both division parameters for the case 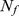 = 41 which corresponds to a high confidence population size. In Fig. 5A we show how the probability of generating 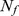 = 41 changes as we vary the parameter 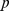 for different 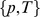 values in the parameter space that yielded this as the most probable 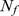 in the previous section (see Fig. 5C for a zoom in on the parameter space selected). For the 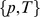 pair that yielded 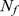 = 41 with the highest confidence (green) we see that it is fairly robust to parameter variation out to about 5% variation. For a 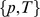 pair that resides near a boundary of a neighbouring 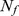
region, the probability of generating 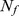 = 41 drops more rapidly and is less robust.

In Fig. 5B, we show the effects of perturbing the cutoff threshold at each division. Again for the most high confidence 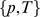 pair the likelihood of generating 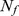 = 41 drops at around 6% variation. However what is striking is that for some lesser confidence 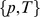 pairs, perturbations in the threshold actually help to improve the likelihood of generating the given population size. Indeed for the 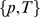 pair on the right boundary of the parameter region, with a threshold variation of ∼ 5% the probability of generating 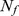 = 41 can be raised from <80% with no variation to >90%. We speculate that this must arise due to some effective cancelation in division errors that increases the fidelity, since individually each 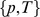 pair for these values of 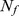 have confidences <90%. In contrast to this and other special population sizes, those that are of lower confidence are less robust to perturbations in 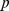 and 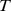 (see Fig. S5).

## Discussion

In this paper we have shown how an hourglass model for an intrinsic cell cycle factor coupled with asymmetric division can produce an arbitrary final cell population size. Such a mechanism has been argued and shown to potentially govern the development and differentiation of certain tissues [8]. Besides being able to generate any final population size, the model also showed that given a final fixed cell count, different parameter choices could produce a variety of lineage trees from linear to non-linear giving flexibility in the differentiation process. Also depending on the degree of noise, these topologies can perform better or worse at generating fixed populations with high confidence. Topology differences in tissue development has been seen in Drosophila neuroblasts, where type I neuroblasts produce linear tree lineages whereas much more proliferative type II neuroblasts have much more non-linear lineages. When we considered the addition of division noise to the model, a number of unique final population sizes were shown to exhibit robustness. Such population sizes were found to be able to be generated with much higher confidence than even nearby sizes. It is intriguing to think that if such an intrinsic timer coupled with asymmetric division is at work one might expect to see biases in the distribution of final population sizes.

Can one find examples of such a mechanism in Nature? As mentioned in the introduction, tissue differentiation usually involves a mix of intrinsic and extrinsic cues guiding the proliferation and differentiation process, convolving their contributions to the final population size. Some potential hints exist that with further testing might show that dilution plus asymmetric division may play some role. For example in Drosophila Type I neuroblasts where a linear cascade of transcription factors governs the proliferation and differentiation process, it is known that overexpression of any TF in the cascade leads to extra proliferation [3]. In particular, overexpressing the gene *hunchback* leads to extra rounds of division [16] turning the type I system into something more like the more proliferative type II neuroblasts. Is this extra proliferation due to a gradual dilution of the extra *hunchback* in the system, prolonging the cascade? In many stem-cell systems, mutations in the factors that cause errors in the partitioning also lead to increased proliferation and tumor like growth [10]. Could this be due to changes in what would be the equivalent of the asymmetry parameter, 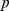 in our model, or in the initial amounts of factor, 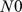
? Further exploration of such systems within the context of the suggested model would be required to assess the degree to which such a mechanism plays any role.

There are other experimental systems where the proposed mechanism may be more directly relevant. The original hourglass model for an intrinsic timer was suggested in the context of oligodendrocyte differentiation [7], [8]. For these stem cells it is a combination of accumulation and dilution of cell cycle regulators (such as the Cdk-inhibitor p27) that regulated proliferation. However the role of the asymmetric division of such factors has not been discussed in great detail for this system. So this system too may only have the proposed mechanism acting in part.

A more likely system for direct testing of the model would be in unicellular organisms. Recent work has shown that the accumulation of misfolded protein in bacteria and yeast may lead to ageing, and reduced cell divisions [10], [17], [18]. Misfolded protein is known to be asymmetrically partitioned between mother and daughter cell [19]. Overexpressing such proteins leads to reduced proliferation as both mothers and daughters undergo fewer divisions. This represents the inverse of our model where instead of dilution a cell-cycle factor is being accumulated. Once the factor is above some threshold, cells can no longer divide. A theoretical model showed that there is a benefit to asymmetrically dividing the accumulation of such deleterious material within an ageing unicellular population [20].

One could also imagine a direct testing of the model using synthetic biology methods with yeast as the model. Budding yeast would allow for the asymmetric partitioning of factors due to unequal volumes of the resulting cells post division. One might imagine generating a mutant defective in one of the constitutively present cell cycle factors such as the cyclin-dependent kinase, Cdk. The lineage and division process could be started through the transient expression of the missing Cdk off of an inducible promoter. After the initial burst of the protein, it would be subsequently diluted asymmetrically through repeated rounds of division. Using automated lineage tracking, it would be possible to track the exact details of the sequence of divisions through to termination.

Lastly we comment on the different active mechanisms for the asymmetric division of biological macromolecules like protein and RNA**.** While these processes are often conceptualized as producing an absolute (all-or-none) asymmetry in the daughter cells, it is plausible to assume that at least a small fraction of these molecules could be inherited by one of the daughter cells. Whether this fraction (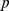 in our model) can be tuned by tweaks in the various parameters of the mechanism remains to be explored experimentally in either natural or synthetic systems. An example of such a parameter that could potentially be ‘tuned’ is the affinity of an mRNA to the protein complexes that are asymmetrically shuttled by myosin motors towards one of the daughter cells [21]. Also reaction-diffusion schemes have been to also play a role where mRNAs are captured at a localizing region, leading to gradients across the cell [11]. Though again, how well these can be manipulated have yet to be explored to our knowledge.

Furthermore, upon experimental verification of the basic scheme hypothesized here, elaborations to the model can be made. Some of these are: the inclusion of production and degradation rates for the factor, interaction between multiple asymmetrically dividing molecules, and coupling between the currently independent division mechanism (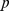) and thresholding mechanism (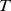). The model also has similarities to branching processes such as those studied in cosmic ray physics, for instance the decay of high energy nucleons into lower energy particles. Analytical solutions using generating functions for such processes have been worked out [22] and it will be interesting to see to what degree they relate to the branching process suggested here. Asymmetric division coupled with dilution should act as a proof of principle for the viability of a feedback-free process for set-point population size control.

## Methods

### Deterministic Simulations

A binary tree was iteratively generated where every node was labeled 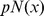 and 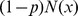 at each branch point, where 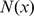 is the label of the node from which they branched and provided that 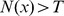. 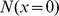 or the number of molecules in the initial ‘cell’ was taken to be 1. This allowed dispensing with a third parameter 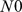 and the simplification of the range of parameter 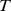 to be between 0 and 1 (i.e. as a fraction of 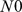). The parameter 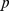 was explored between 0 and 0.5. Both of these parameters were explored with a mesh size of 0.0025.

### Stochastic Simulations

Due to the impact of the initial number of molecules, 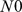, on the size of binomial noise at each division it therefore had to be considered as a third parameter to the system. The simulations were carried out at different 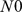 for all the different values of 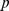 and 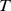 simulated in the deterministic case. At each division the values for the labels of the two cells were drawn binomially from the number of molecules in the parent cell with probability 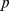 and 1-*p*. For each 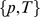 value simulations were carried out 100 times.

### Defining Topologies

The linear topologies were defined as those where the population size at each iteration time point during the growth of the tree, i.e. 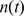, followed 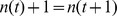, for the whole course of growth until the final size was reached. Figure 1A is an example of such a topology. Non-linear topologies like that in Figure 1B, were defined as those which do not satisfy this relationship for even a single value of t.

### Parameter Perturbations

The simulations were carried out as in the stochastic simulations, except that at every division the values for 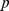 and 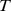 were chosen from a Gaussian distribution. The mean of this distribution was equal to the value of 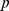 or 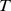 in the parameter space that was being explored, and the standard deviation of the distribution ranged from 0 to 10% of the mean. For each value of 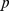, 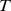, and sigma, 1000 simulations were carried out.

## Supporting Information

Figure S1
**The variety of topologies as a function of 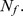
** The number of unique topologies as measured by the length of time taken for 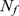 to be reached. Note that it peaks around 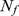 = 50. This is due to the counteracting forces of increased variety due to increase in 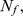 and decreased number of 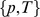 pairs that give a certain 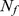 with increase in 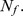

(PDF)

Figure S2
**Difference between deterministic and stochastic results.** The difference between 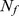 from deterministic simulations and the most probable 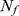 from the stochastic simulations is plotted as a function of 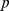 and 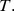 The difference is highest on the border between regions of different 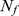 and where 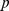 and 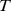 are close to 0 (where 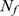 is larger). The identity between the stochastic and deterministic results (measured as a percentage of all the 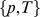 values explored) approaches 100% with increase in 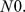

(PDF)

Figure S3
**The most probable 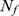 as a function of 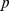 and 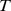.** The mode of the distribution of 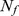 is plotted as a function of 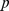 and 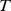 when 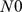 = 10,000.(PDF)

Figure S4
**Relationship between topology and confidence at different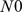.** At smaller values of 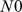 non-linear topologies tend to yield 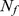 with higher confidence.(PDF)

Figure S5
**Perturbations of low-confidence parameter combinations.** Perturbations of non-special 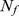 (i.e. those for which there were no high-confidence parameter combinations) result in rapid decay in the confidence, and no critical behavior. 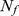 = 40 and 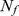 = 23 are chosen to compare with 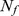 = 41 in Figure 5 (main text).(PDF)
