# Supplementary figures and images for: A Model for Cell Population Size Control Using Asymmetric Division
Source: PLoS One. 2013 Sep 5;8(9):e74324. doi: 10.1371/journal.pone.0074324 (PMC3764109; doi:10.1371/journal.pone.0074324)

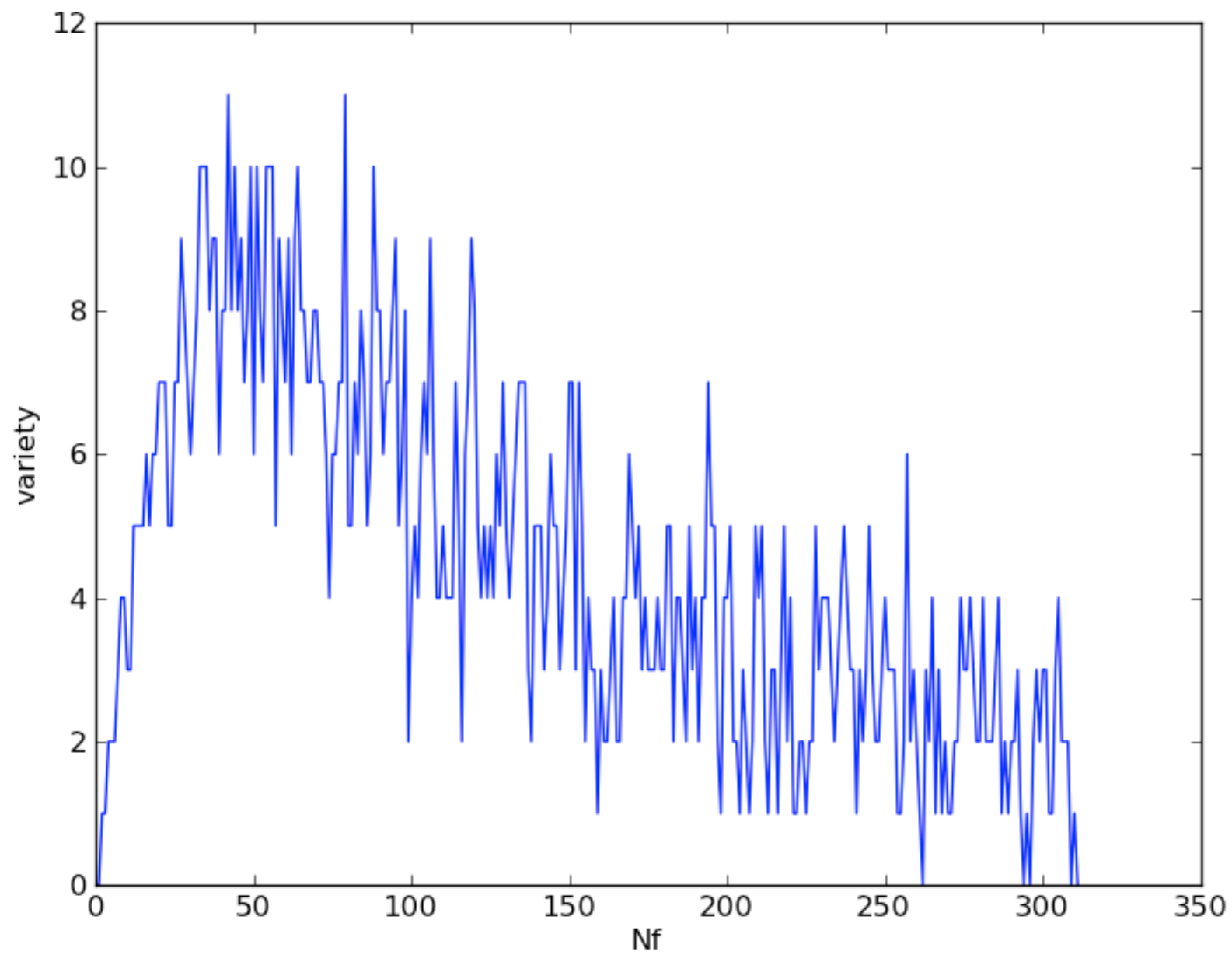

Supplement: Figure S1 — The variety of topologies as a function of The number of unique topologies as measured by the length of time taken for to be reached. Note that it peaks around = 50. This is due to the counteracting forces of increased variety due to increase in and decreased number of pairs that give a certain with increase in (PDF) [file pone.0074324.s001.pdf]

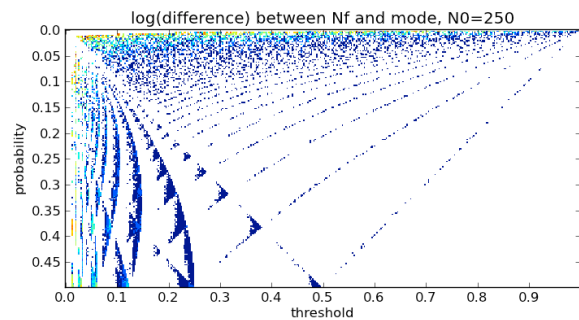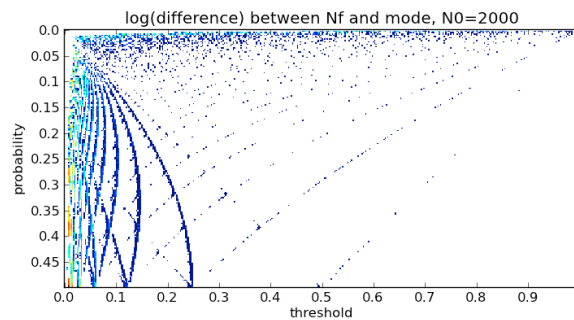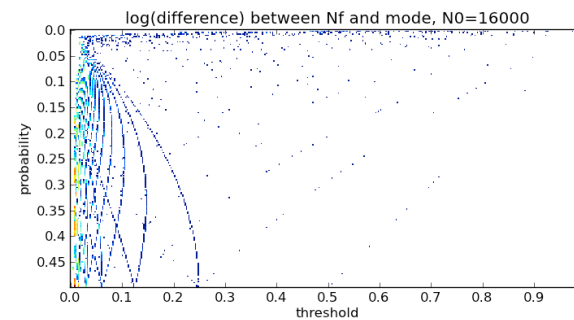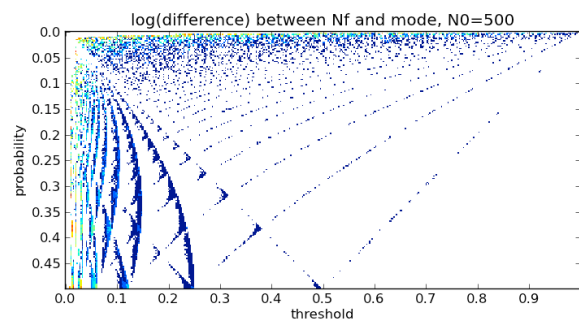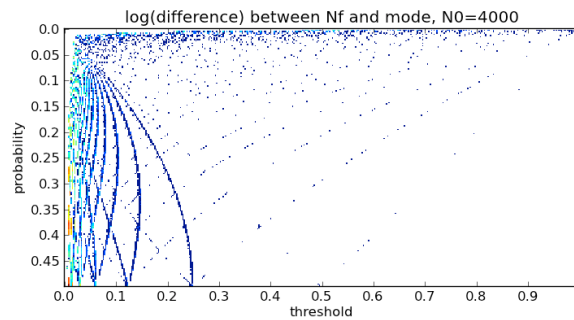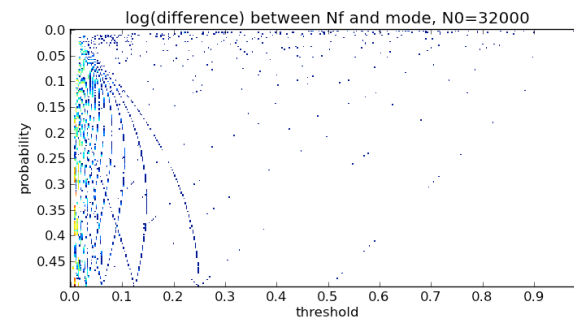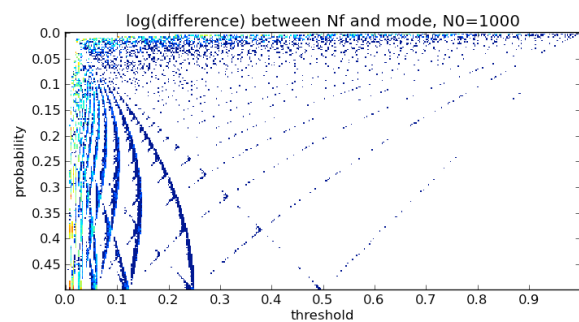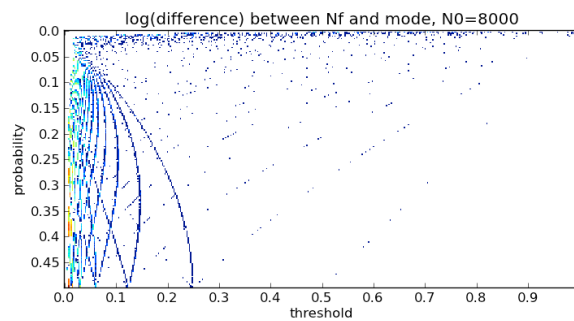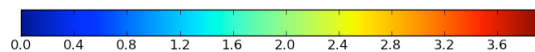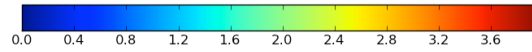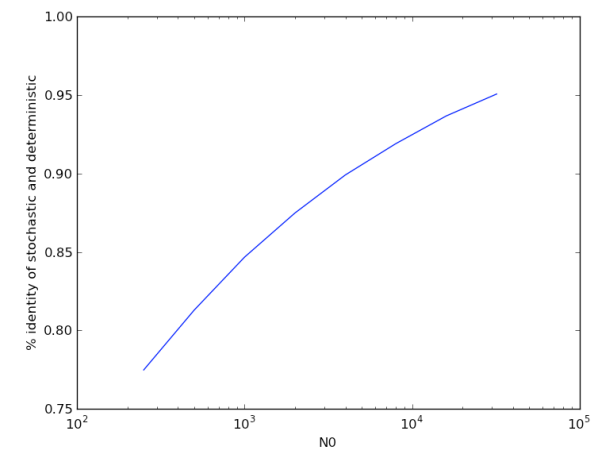

Supplement: Figure S2 — Difference between deterministic and stochastic results. The difference between from deterministic simulations and the most probable from the stochastic simulations is plotted as a function of and The difference is highest on the border between regions of different and where and are close to 0 (where is larger). The identity between the stochastic and deterministic results (measured as a percentage of all the values explored) approaches 100% with increase in (PDF) [file pone.0074324.s002.pdf]

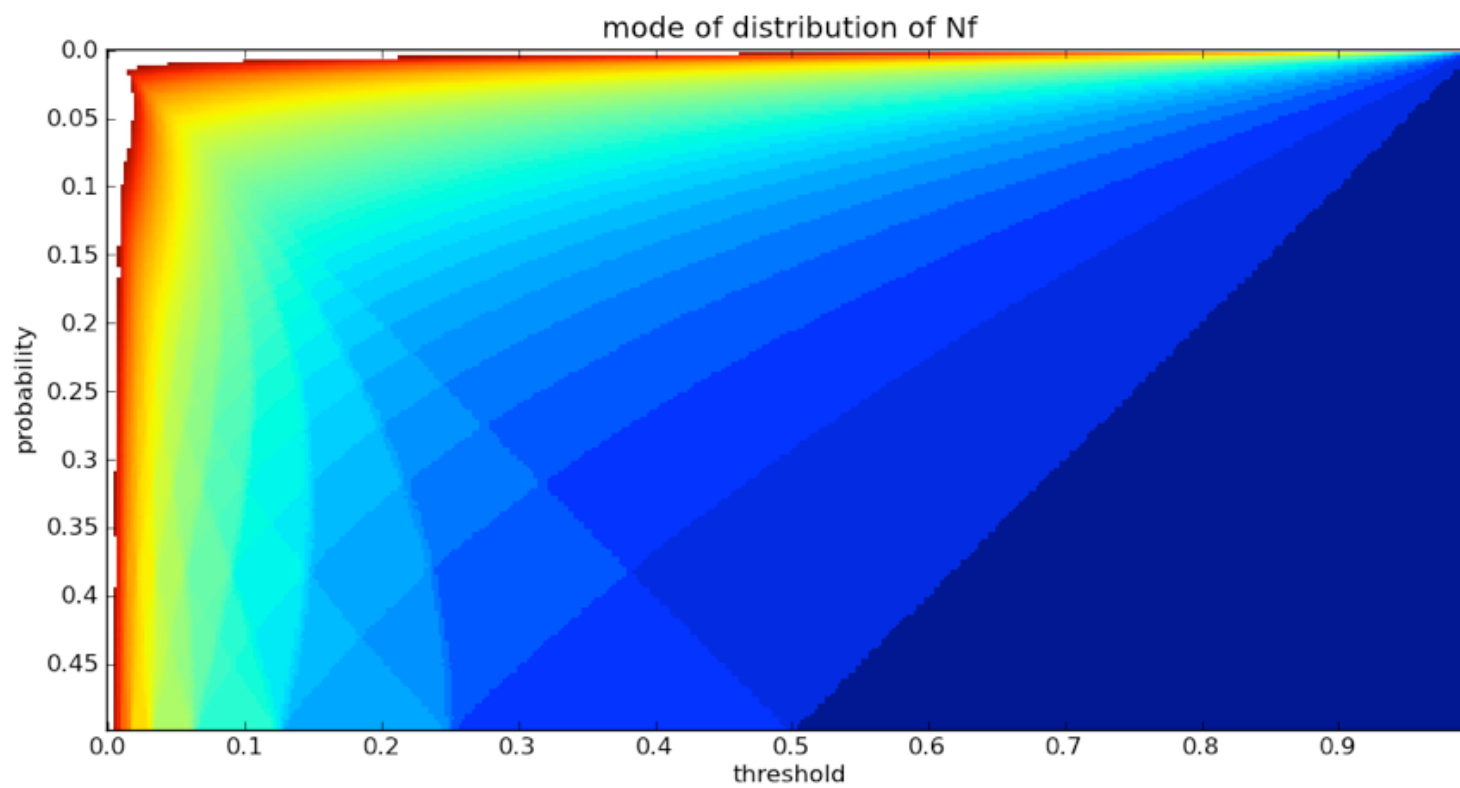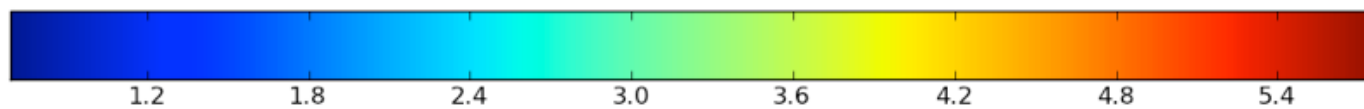

Supplement: Figure S3 — The most probable as a function of and . The mode of the distribution of is plotted as a function of and when = 10,000. (PDF) [file pone.0074324.s003.pdf]

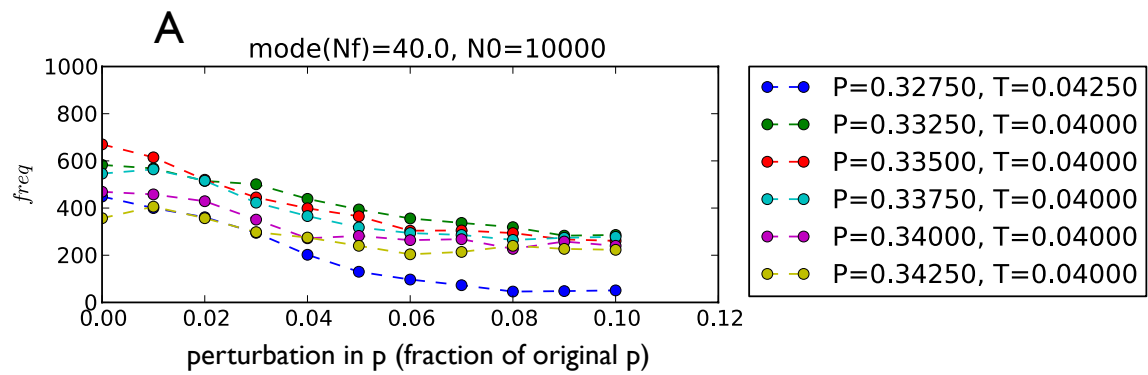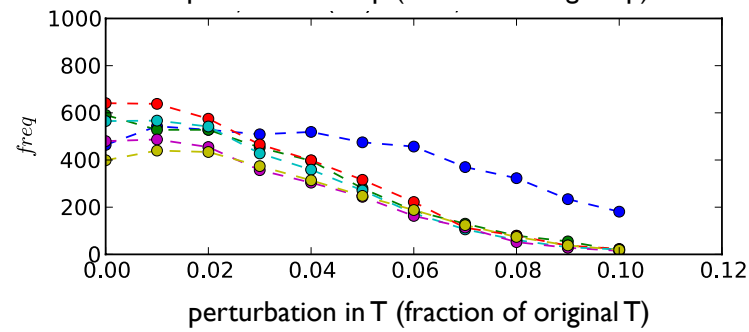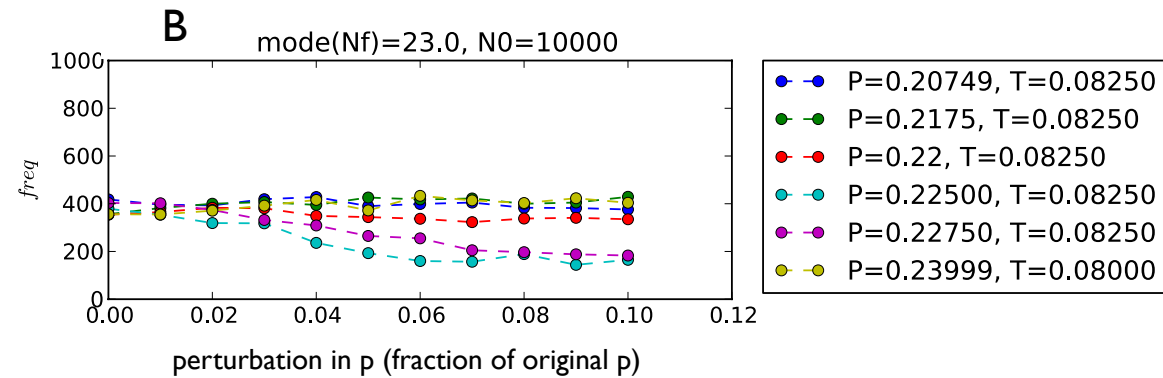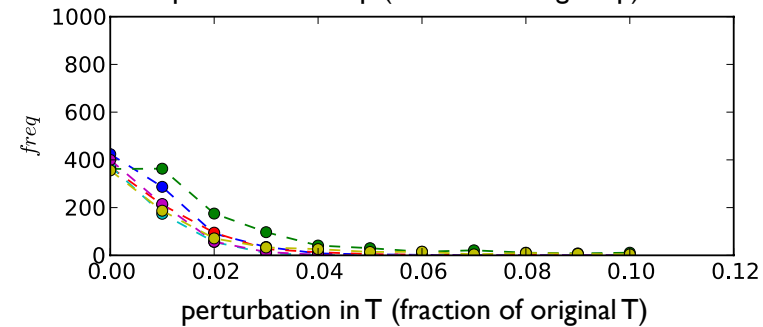

Supplement: Figure S5 — Perturbations of low-confidence parameter combinations. Perturbations of non-special (i.e. those for which there were no high-confidence parameter combinations) result in rapid decay in the confidence, and no critical behavior. = 40 and = 23 are chosen to compare with = 41 in Figure 5 (main text). (PDF) [file pone.0074324.s005.pdf]
