# Supplementary material for: A Model for Cell Population Size Control Using Asymmetric Division
Source: PLoS One. 2013 Sep 5;8(9):e74324. doi: 10.1371/journal.pone.0074324 (PMC3764109; doi:10.1371/journal.pone.0074324)
Supplement: Figure S4 — Relationship between topology and confidence at different. At smaller values of non-linear topologies tend to yield with higher confidence. (PDF) [file pone.0074324.s004.pdf]

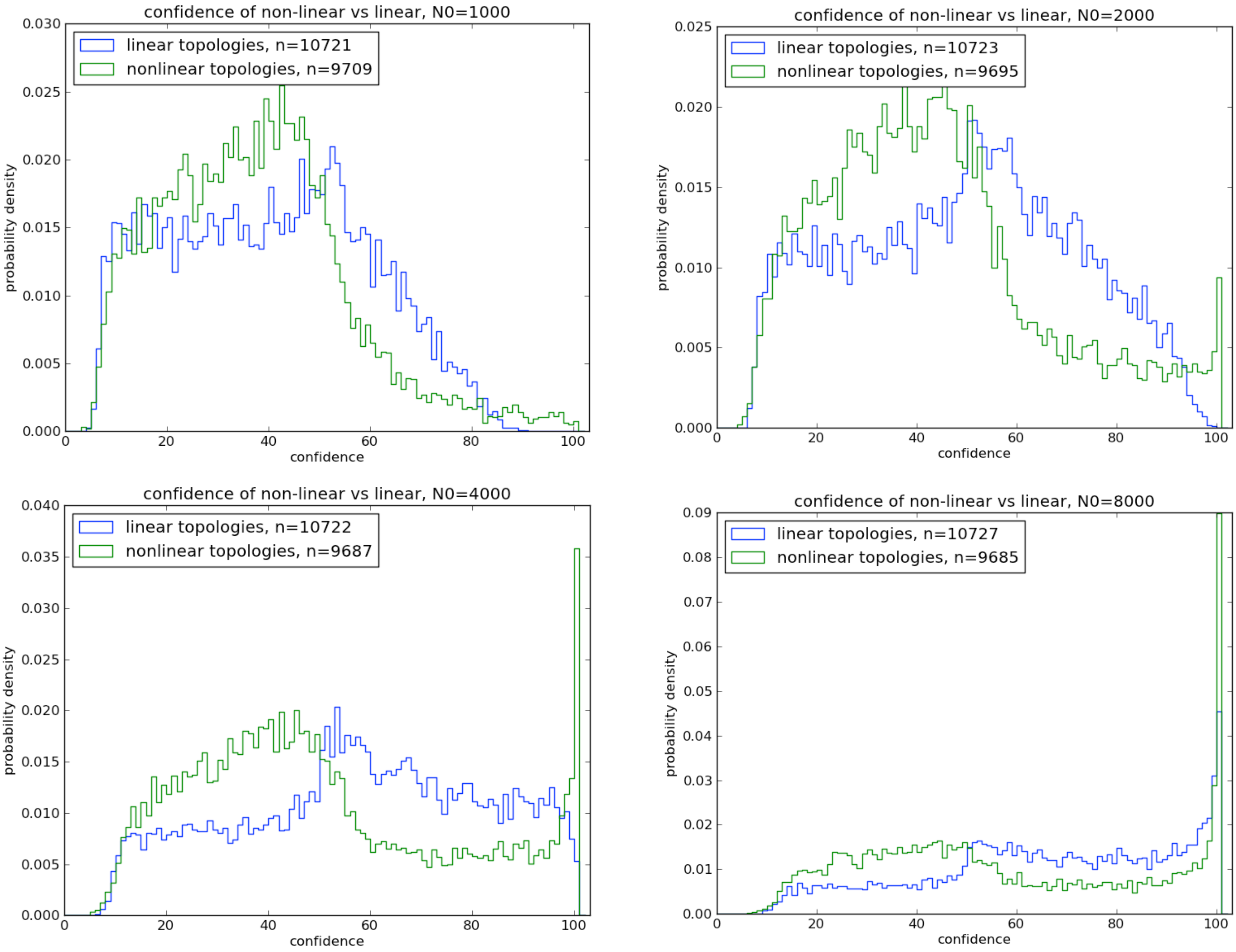
confidence of non-linear vs linear, N0=1000
linear topologies, n=10721
nonlinear topologies, n=9709
probability density
confidence
confidence of non-linear vs linear, N0=2000
linear topologies, n=10723
nonlinear topologies, n=9695
probability density
confidence
confidence of non-linear vs linear, N0=4000
linear topologies, n=10722
nonlinear topologies, n=9687
probability density
confidence
confidence of non-linear vs linear, N0=8000
linear topologies, n=10727
nonlinear topologies, n=9685
probability density
confidence
